# Flavonoid compound icariin enhances BMP-2 induced differentiation and signalling by targeting to connective tissue growth factor (*CTGF*) in SAMP6 osteoblasts

**DOI:** 10.1371/journal.pone.0200367

**Published:** 2018-07-10

**Authors:** Bing Xu, Xueqiang Wang, Chengliang Wu, Lihe Zhu, Ou Chen, Xiaofeng Wang

**Affiliations:** 1 Integrated Traditional Chinese and Western Medicine Hospital of Wenzhou Affilated Hospital of Zhejiang Chinese Medicine University, Zhe Jiang, China; 2 Department of Sport Rehabilitation, Shanghai University of Sport, Shanghai, China; 3 Zhejiang Chinese Medical University, Hangzhou, Zhejiang, China; Università degli Studi della Campania, ITALY

## Abstract

**Background:**

Icariin, a major active flavonoid glucoside, is widely used for the treatment of bone injury and rebuilding in the clinic because of its roles in suppressing osteoblastogenesis and promoting osteogenesis. The senescence-accelerated mouse SAMP6 was accepted as a useful murine model to reveal the mechanism of senile osteoporosis and the therapeutic mechanism of drug activity. However, little is known about the characteristics of SAMP6 osteoblasts and the associated regulatory roles of icariin.

**Methods:**

We isolated and cultured osteoblasts from SAMP6 or SAMR1 mice and compared their proliferation, migration, and differentiation by performing the CCK-8 assay, cell counting assay, EdU staining, cell cycle analysis, ALP staining and activity measurement, Alizarin red staining, and RT-qPCR analysis to measure the levels of osteoblast markers, including RUNX2, Colla1 and Oc. To assess the effects of icariin on BMP-2-induced osteoblast differentiation, after BMP-2 treatment, osteoblast markers were analyzed by RT-qPCR and semi-quantitative Western blotting. The effects of icariin on connective tissue growth factor (*CTGF*) were measured by RT-qPCR. shRNA targeting *CTGF* mRNA was employed to knockdown its expression level in osteoblasts.

**Results:**

The SAMP6 osteoblasts presented decreased the development and differentiation activity compared with SAMR1 osteoblasts, indicating that they are the potential mechanisms underlying age-associated disease. Moreover, SAMP6 osteoblasts presented upregulated *CTGF* compared with SAMR1 osteoblasts. Icariin enhanced BMP-2-induced osteoblast differentiation by downregulating *CTGF* expression, which tightly regulates osteoblast differentiation. By downregulating *CTGF*, icariin treatment upregulated phosphate-Smad1/5/8, indicating its activating effects on the BMP signaling pathway.

**Conclusion:**

These results suggest that decreased osteoblast development and function potentially contributes to age-associated disease. Icariin exerts enhancing effects on BMP-2-mediated osteoblast development via downregulating *CTGF*.

## Introduction

Senescence-accelerated mice (SAM) are a series of inbred stains developed from the AKR/J strain consisting of 4 senescence-resistant strains (SAMR) and 9 senescence-prone strains (SAMP) [1, 2]. Compared with the SAMR strains, which present normal senescence, SAM-P6 (SAMP6) strains exhibit accelerated-senescence phenotypes by developing osteoporosis within a few months of birth [3], increased numbers of mature adipocytes (Aps) within the bone marrow [4] or decreased osteoblastogenesis [5], and they have been accepted as an experimental animal model to research age-associated disease [6], including being used as a murine model of senile osteoporosis. By employing the SAMP6 model, O’Sullivan and colleagues found that one of the main factors contributing to the accelerated loss of bone mass in SAMP6 is the presence of impaired osteoblast progenitors affecting proliferation and cell viability in SAMP6 bone marrow, indicating the potential mechanism of senile osteoporosis [7]. However, little is known about the contribution of SAMP6 osteoblasts to the age-associated phenotype.

Icariin is an active compound extracted from the Traditional Chinese Herb (TCH) Epimedium brevicornum Maxim. Icariin was reported to exert therapeutic effects on the kidney, joints and other disorders by demonstrating bone-protective actions [8–10]. In postmenopausal women, icariin treatment stimulated bone formation and increased bone mass [9]. By exposing bone marrow stromal cells to icariin, the marrow contained osteoblast progenitors presented upregulated expression levels of various proteins critical to bone matrix deposition, including runt-related transcription factor 2 (RUNX2), alkaline phosphatase (ALP), collagen type I alpha 1 (Colla1) and osteocalcin (Oc), which are required for osteoblast development and function [11–14]. By contrast, icariin inhibited the formation and activation of osteoblasts [15, 16], a finding that is consistent with the protective effects on bone mass. Icariin is also reported to regulate osteoblasts directly. Zhang et al. reported that icariin treatment promoted proliferation and bone formation by upregulating RUNX2, ALP, Colla1 and Oc after bone morphogenetic protein-2 (BMP-2) stimulation [17]. BMP-2 promotes osteogenesis through the canonical Smad pathway involving Smads 1, 5 and 8. After phosphorylation by BMP-2 exposure, p-Smad1/5/8 transcriptionally activates downstream target genes, such as Runx2 [18, 19]. However, whether icariin could regulate SAMP6 or SAMR1 osteoblasts to affect proliferation, differentiation and mineralization remains unclear.

Connective tissue growth factor (*CTGF*), which is expressed and secreted by osteoblasts during proliferation, differentiation, bone formation and fracture healing, has been shown to regulate osteogenesis in osteoblasts [20, 21]. Kawaki et al. found that knockout of *CTGF* in osteoblasts delayed osteoblast maturation and mineralization in cultured osteoblasts [22]. Lambi et al. reported that upregulation of *CTGF* inhibited the expression of RUNX2, Colla1 and Oc and resulted in the inhibition of proliferation, differentiation and mineralization in osteoblasts [23]. Overexpression of *CTGF* decreased the protein levels of phosphorylated Smad1/5/8, suggesting that *CTGF* regulates the BMP signaling pathway [24]. All these data indicate that appropriate expression is important for *CTGF* as a regulator of osteoblast development and function.

In this study, our objective was to compare the differences between SAMP6 osteoblasts and SAMR1 osteoblasts and potential regulatory roles of icariin in the physiological processes of SAMP6 osteoblasts such as proliferation, migration, differentiation and mineralization. In addition, the mechanism underlying the promotion effects of icariin on osteoblast development and function was demonstrated. Because of the difference in osteoblastogenesis in SAMP6, we hypothesized that SAMP6 osteoblasts have a diminished response to BMP-2, which is reversed by icariin treatment.

## Material and methods

### Primary osteoblast cell culture

All animal experiments were approved by the Wenzhou Traditional Chinese Medicine Hospital Ethics Committee. New born SAMP6 and SAMR1 mice were bought from Chengdu Dashuo Experimental Animal Research Center (Chengdu, China) and were allowed to be free access to food and water and kept under standardized environmental conditions (12h light/dark cycle, 24°C±1°C and 55%±1% relative humidity). Animals were sacrificed to isolate primary osteoblasts from parietal calvaria pieces in accordance with the Guide for the Care and Use of Laboratory Animals. Briefly, after being anaesthetized using combination of 100mg/kg ketamine and 25mg/kg xylazine, and then decapitated, calvaria pieces were placed in digestion media containing 0.1% Collagenase (Sigma–Aldrich, St. Louis, MO, USA)/1% trypsin (Life Technologies, Grand Island, NY, USA) for 30 min at 37°C. Digested supernatant was centrifuged at 400g, 4°C for 10 min and cells were resuspended with DMEM (Life Technologies) supplemented with 10% fetal bovine serum (FBS; Life Technologies) and plated in 100 mm dishes (Corning Incorporated Life Science, NY, USA). For Icariin (Chinese National Institute for Control of Pharmaceutical and Biological Products, Beijing) treatment, 10^−7^, 10^−6^, or 10^−5^ M of Icariin was supplemented to medium for 7, 14, or 21 days. For BMP-2 stimulation, recombinant BMP-2 (R&D systems, Minnesota, USA) was reconstituted to a concentration of 10μg/ml in PBS containing 0.1% BSA and stored at -20°C. For stimulating differentiation, the final concentration of BMP-2 (100ng/ml) was used every three days. For evaluating levels of p-Smad 1/5/8, cells were pre-cultured in 0.1% FBS for 24 hrs. and then stimulated with BMP-2 for 2, 4, or 6 hrs.

### Detection of cell proliferation by CCK-8 assay

SAMP6 or SAMR1 osteoblasts were seeded into 96-well plates (100μL/well; 5000 cells/well) and allowed to attach overnight. At 1, 2, 3, 4 and 5 days, 10μL of CCK-8 solution (Sigma–Aldrich, St. Louis, MO, USA) was added into each well for 4-hour incubation at 37°C in 5% CO_2_ incubator. The absorbance of each sample was measured at a wavelength of 620 nm. The experiments were done three times.

### Growth curve by cell counting

SAMP6 or SAMR1 osteoblasts were seeded into 12-well plates (1mL/well; 10000 cells/well) and allowed to attach overnight. Cells were maintained at 37°C in 5% CO2 incubator. For each day, cells were suspended using trypsin (Life Technologies, Grand Island, NY, USA) and then counted. The average was obtained. The experiments were done three times. Cell doubling time were calculated using GraphPad Prism v5.0 software for Windows (GraphPad Software Inc., San Diego, CA) by nonlinear regression (exponential growth equation) analysis.

### Proliferation assay by EdU staining

Cultured cells were stained with EdU detection kit (Ribobio, Guangzhou, China) following the manufacturer’s instruction. Briefly, cells were incubated with 50 μM EdU labeling medium at 37°C for 2h in 5% CO_2_ incubator. After immobilization using 500 μL 4% paraformaldehyde at room temperature for 10 min, cells were stained with Apollo^®^567 solution and Hoechst33342 solution. Cells were observed under a X71 (U-RFL-T) fluorescence microscope (Olympus, Melville, NY). EdU-positive cells represent proliferating cells and Hoechst33342-positive cells represent total cells.

### Cell cycle analysis

Cultured cells were fixed with ice-cold 70% ethanol overnight, cells were stained with 50 μg/ml propidium iodide (PI, Sigma–Aldrich, St. Louis, MO, USA) and analyzed by Navios flow cytometers (Beckman Coulter, Brea, CA, USA).

### Staining and activity measurement of ALP

Osteoblasts were cultured for 14 and 21 days with or without 100ng/ml BMP-2. For ALP staining, cells were fixed with 4% paraformaldehyde at room temperature for 10 min according to previous report [25]. For ALP activity analysis, cells were washed three times with ice-cold PBS and digested with 0.25% trypsin and suspended. Pelleted cells were resuspended in extraction lysis buffer containing 50 mM Tris-HCl 8.0, 150 mM NaCl, 0.5 mM EDTA, 0.25% Nonidet P(NP)-40 and incubated for 30 min at 4°C. By centrifuging at 12000g for 10min, supernatant was collected and total protein amount was quantified by BCA method (Pierce, USA). ALP activity assay detection kit (Nanjing Jiancheng Biotechnology Co. Ltd, Nanjing, China) was employed and the absorbance was measured at 520 nm using microplate reader (Synergy 2 Multi-Mode Microplate Reader; BioTek, Winooski, VT, USA).

### Alizarin red staining

For evaluating mineralization of cultured cells, alizarin red staining was performed. Briefly, cells were washed in HBSS and fixed in 4% ice-cold paraformaldehyde for 10 min at room temperature. Fixed cells were washed with PBS and stained with 40 mM Alizarin Red S (Sigma–Aldrich, St. Louis, MO, USA) for 15 min at room temperature. Cells were washed, air dried and imaged using Olympus-CX41 (Olympus, Japan). After imaging, induced mineralization were quantified by dissolving the bound stain using cetylpyridinium chloride monhdrate (Sigma–Aldrich, St. Louis, MO, USA) and the absorbance at 550 nm was measured on a multifunctional microplate reader (FlexStation 3, Molecular Devices, USA).

### Real-time PCR

Total RNA was extracted from cultured cells using TRIzol (Life Technologies, Grand Island, NY, USA) following the manufacture’s instruction. 1μg of total RNA was used for reverse transcription using an RT-for-PCR kit (Qiagen, Valencia, CA, USA). Real-time PCR was performed with SYBR Green PCR Master Mix (Life Technologies, Grand Island, NY, USA). The PCR cycle was as follows: 95°C for 5 min, 40 cycles of 95°C for 10s, 60°C for 50s. The amplification and analysis were performed using an ABI Prism 7500 Real-Time PCR System (Applied Biosystems, Foster City, CA, USA). Samples were compared using the relative CT method normalized to a housekeeping gene using 2^-△△CT^ [26]. The following primer sequences were used: RUNX2 forward primer, 5’-GACTGTGGTTACCGTCATGGC-3’ and revere primer, 5’-ACTTGGTTTTTCATAACAGCGGA-3’; Colla1 forward primer, 5’-GCTCCTCTTAGGGGCCACT-3’ and reverse primer, 5’-ATTGGGGACCCTTAGGCCAT-3’; OC forward primer, 5’-TGCTTGTGACGAGCTATCAG-3’ and reverse primer, 5’-GAGGACAGGGAGGATCAAGT-3’; JMJD3 forward primer, 5’-TGAAGAACGTCAAGTCCATTGTG-3’ and reverse primer, 5’-TCCCGCTGTACCTGACAGT-3’; IGFBP-2 forward primer, 5’-CAGACGCTACGCTGCTATCC-3’ and revere primer, 5’-CCCTCAGAGTGGTCGTCATCA-3’; *CTGF* forward primer, 5’-GGCCTCTTCTGCGATTTCG-3’ and reverse primer, 5’-GCAGCTTGACCCTTCTCGG-3’; β-actin forward primer, 5’-GTGACGTTGACATCCGTAAAGA-3’ and reverse primer, 5’-GCCGGACTCATCGTACTCC-3’.

### Design and introduction of shRNA against mouse *CTGF*

The shRNA-1 against mouse *CTGF* (sh-*CTGF*-1; 5’- AAGACCTGTGCCTGCCATTACAACTCTTGATTGTAATGGCAGGCACAGGTGAGGT-3’), shRNA-2 against mouse *CTGF* (sh-*CTGF*-2; 5’- AAAGAGTGGAGCGCCTGTTCTAACTCTTGATTAGAACAGGCGCTCCACTCTGAG-3’) or Scrambled shRNA sequence (shScrambled; 5’-AAGCATATGTGCGTACCTAGCATCTCTTGAATGCTAGGTACGCACATATGCGAGGT-3’) were cloned to the multiple cloning sites of a eukaryotic expression vector, GV102 (GeneChem, Shanghai, China).

One day prior to transfection of plasmid-shRNA, cells were seeded at 50% confluency on 6-well tissue culture plate. Following the manufacturer’s recommendation, 1.6 μg sh-*CTGF*-1, sh-*CTGF*-2 or sh-*CTGF*-1/2 mixed equally with sh-*CTGF*-1 and sh-*CTGF*-2 was mixed with Lipofectamine LTX Reagent (Invitrogen, California, USA) to each well. 4 hour later, medium was refreshed. 24 or 48 hour later, transfected cells were performed for the following assays.

### Migration assays

Migration was evaluated by scratch assay and transwell assay. For scratch assay, cultured cells were seeded into 12-well plate and incubated at 37°C and 5% CO_2_ overnight. A 200 μL pipette tip was used to obtain the cell free lane. A ruler was used as a guide to obtain a straight line. Images were taken at 0, 12 and 24 h using a X71 (U-RFL-T) fluorescence microscope (Olympus, Melville, NY). For transwell assay, 5×10^4^ cells were seeded in the upper chambers, and 600 μl DMEM containing 10% FBS was added to the lower chamber. After incubation at 37°C for 12 and 24 h, cells were fixed using 4% paraformaldehyde at room temperature for 10 min and stained in 0.5% crystal violet at room temperature for 10 min. After three washes in PBS, images were taken.

### Colony formation

1000 cells were seeded in 12-well culture plates in a 5% CO_2_ incubator for 14 and 21 days. Cells were fixed in 4% paraformaldehyde for 10 min at room temperature and stained with 0.5% (w/v) crystal violet for 10 min at room temperature. The experiments were performed in triplicate.

### Western blot

Cells were digested using trypsin and collected. Cells were incubated with extraction lysis buffer containing 50 mM Tris-HCl 8.0, 150 mM NaCl, 0.5 mM EDTA, 0.25% Nonidet P(NP)-40 and incubated for 30 min at 4°C. After centrifugation at 12000g, 4°C for 10min, supernatant was collected and fractionated by 10% SDS-PAGE gel. After electrophoresis, blots were transferred to PVDF membrane (Life Technologies, Grand Island, NY, USA). The membrane was blocked with PBS containing 5% BSA (Sigma–Aldrich, St. Louis, MO, USA) for 1 h and incubated with the following primary antibodies: anti-osteocalcin (1:2000; Abcam), anti-Collagen I (1:1000; Abcam), anti-RUNX2 (1:1000; Abcam), anti-*CTGF* (1:1000; Abcam), anti-total Smad1/5/8 (T-Smad1/5/8; 1:1000; Abcam), anti-phospho Smad1/5/8 (p-Smad1/5/8; 1:1000; Abcam) or anti-β actin (1:4000; Abcam) at 4°C overnight. After three washes with PBS, membrane was incubated with horseradish-peroxidase (HRP) conjugated donkey anti-rabbit (1:10000; Abcam) for 2 h at room temperature. The membrane was incubated with SuperSignal West Pico Chemiluminescent Substrate (Thermo Scientific, Waltham, MA, USA), and exposed to film.

### Statistical analysis

Statistic comparison between results from multiple groups were analyzed using one-way ANOVA followed by Dunnett’s test. For experiments involving two groups, an unpaired Student’s t-test was performed. A value of *P* < 0.05 was considered statistically significant. Data are expressed as mean ± SD. A P-value < 0.05 was considered statistically significant.

## Results

### Osteoblasts derived from SAMP6 mice display decreased cell proliferation and migration but not differentiating capacity compared with those derived from SAMR1 mice in vitro

**Fig 1:**
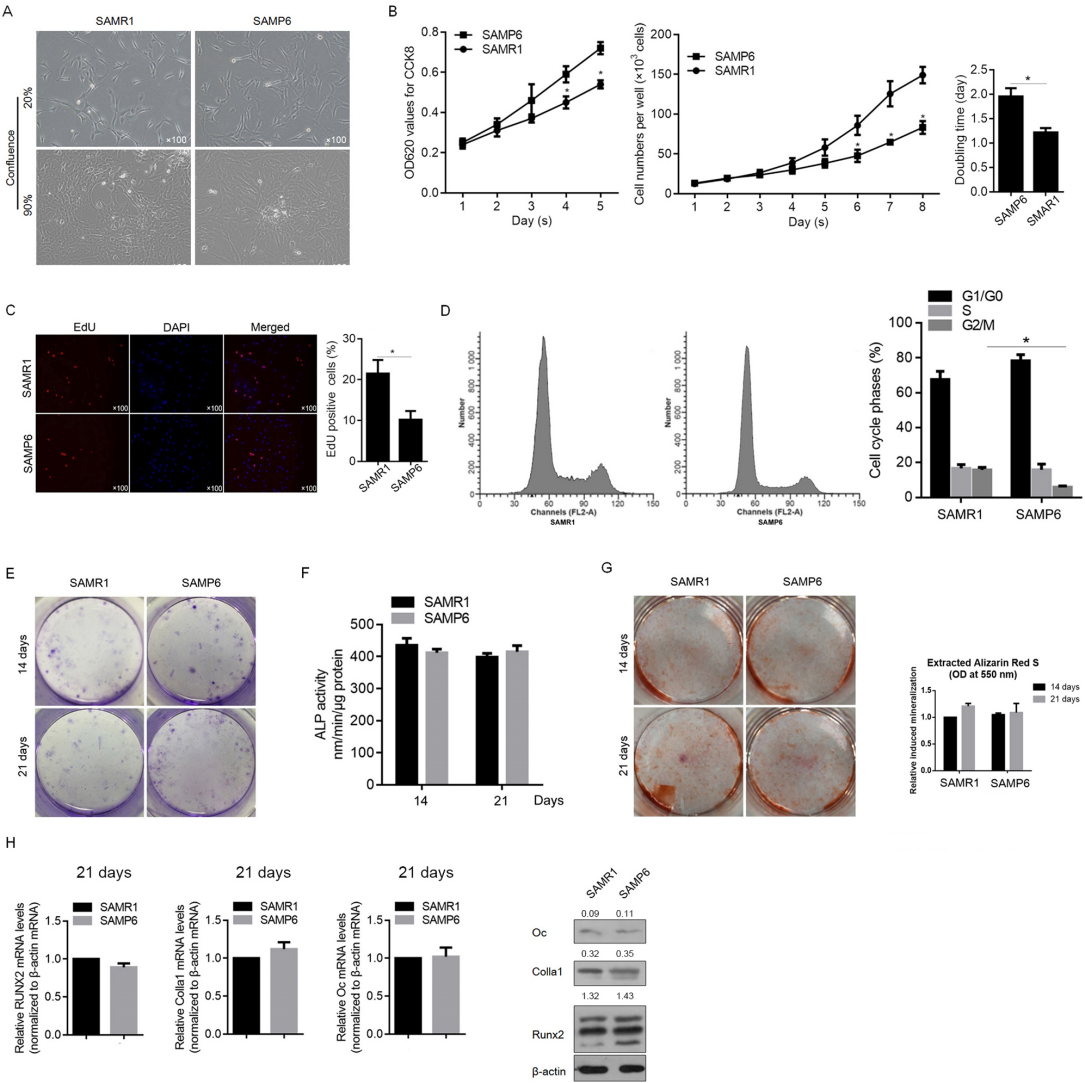
Osteoblasts in SAMP6 mice presented weaker cell proliferating activity than SAMR1. (A) Morphology of SAMR1 and SAMP6 osteoblasts. (B) The CCK-8 assay was performed to detect the cell viability of osteoblasts from SAMP6 or SAMR1 mice. *P<0.05 vs. SAMR1. (C) EdU staining was performed to detect proliferating osteoblasts from SAMP6 or SAMR1 mice. (D) PI staining was performed to analyze the distribution of the cell cycle phases of osteoblasts from SAMP6 or SAMR1 mice. Osteoblasts were cultured for 14 or 21 days and were stained for ALP (E) or assayed for ALP activity (F). *P<0.05 vs. SAMR1. (G) Alizarin red S staining was performed to detect the mineralization of osteoblasts. The quantitative data was presented at right panel. (H) The mRNA and protein levels of *Runx2*, *Colla I* and *Oc* were analyzed by RT-qPCR (left panel) and semiquantitative Western blotting (right panel).

To compare the proliferating activity of osteoblasts cultures derived from SAMP6 or SAMR1 mice that showed no detectable morphological difference (Fig 1A), we assessed cell viability using the CCK-8 assay and counted cell number. As shown in Fig 1B, SAMP6 osteoblasts presented an obvious decrease in cell viability and cell number compared with SAMR1 osteoblasts. Moreover, cell doubling time analysis revealed that SAMP6 osteoblasts (1.952 days) took more time to double compared with SAMR1 osteoblasts (1.215 days, Fig 1B). After culturing for 1, 5 and 10 days, the proliferation activity of SAMR1 or SAMP6 osteoblasts was measured by EdU staining. As shown in Fig 1C, the average percentage of EdU-positive cells in SAMP6 osteoblasts revealed a statistically significant decrease compared with that in SAMR1 osteoblasts (*P*<0.05). Because EdU was incorporated into replicating cells, SAMP6 osteoblasts were suggested to present weaker proliferating activity than SAMR1 osteoblasts. To further confirm whether a change in cell cycle progression was the cause of the decreased proliferating activity in SAMP6 osteoblasts, we performed PI staining followed by flow cytometry analysis, and the results showed that SAMP6 osteoblasts obviously increased the proportion of G_0_/G_1_ phase cells and significantly decreased the proportion of G_2_/M phase cells, compared to SAMR1 osteoblasts, without disturbing distribution of S phase (Fig 1D), which potentially contributes to its relative slow proliferating rate.

Alkaline phosphatase (ALP) is one of the earliest markers of osteoblasts differentiation. When we evaluated osteoblast maturation at Day 14 and 21 by ALP staining and activity, there was no detectable difference between SAMP6 and SAMR1 osteoblasts (Fig 1E and 1F). Next, we examined osteoblast mineralization at day 21 by alizarin red staining. Consistent with the ALP activity, no detectable difference was found in mineralization between SAMP6 and SAMR1 osteoblasts (Fig 1G). In the process of osteoblast differentiation, the upregulation of the transcription factor Runx2 is essential and is known to upregulate other critical genes important for later stages of osteoblast differentiation, such as Colla1 and Oc, which are important for the maturation and mineralization phases, respectively [27]. Thus, we measured Runx2, Colla1 and Oc mRNA expression levels in SAMP6 and SAMR1 osteoblasts at days 21. Although SAMP6 osteoblasts showed a decrease in Runx2, Colla1 and Oc mRNA, they are not significant (Fig 1H). These data demonstrate that SAMP6 osteoblasts display an undetectable difference in differentiation under normal osteogenic culture conditions.

### SAMP6 osteoblasts exhibit decreased maturation and mineralization in the presence of BMP-2 compared with SAMR1 osteoblasts

**Fig 2:**
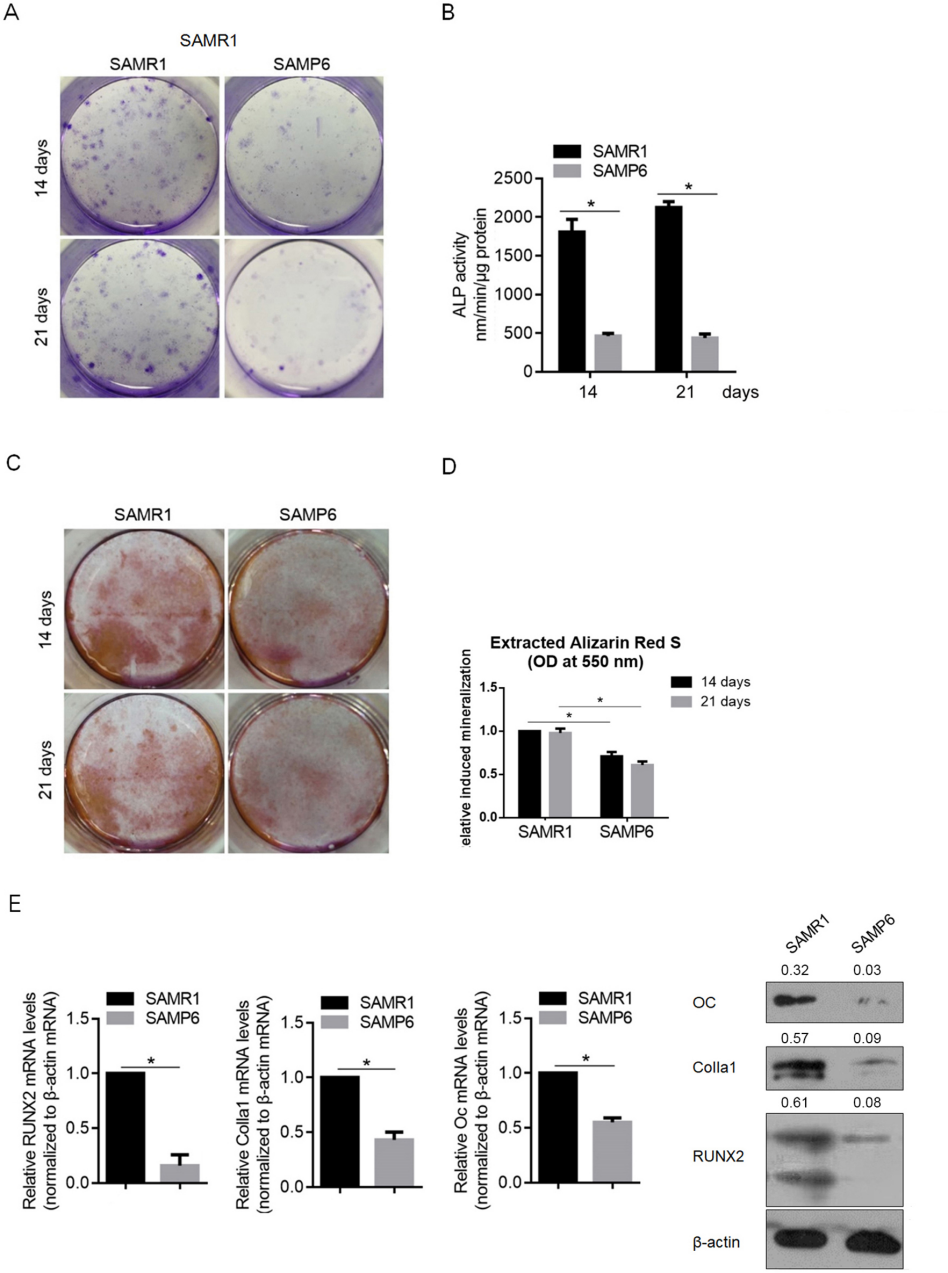
SAMP6 osteoblasts exhibit decreased maturation and mineralization in the presence of BMP-2 compared with SAMR1 osteoblasts. To evaluate the effects of BMP-2 on the maturation and mineralization of SAMP6 or SAMR1 osteoblasts, after 14- and 21-day exposure to BMP-2, the expression levels of ALP (A), ALP activity (B), Alizarin red S staining (C&D) and expression levels of Runx2, Colla and Oc (E) were measured. *P<0.05 vs. SAMR1.

Bone morphogenetic proteins (BMPs) are the most potent endogenous regulator of osteoblast differentiation, and BMP-2 is widely used to stimulate bone regeneration in clinical applications. To figure out whether SAMP6 osteoblasts display different sensitivity to BMP-2 in vitro, we treated osteoblast cultures with recombinant BMP-2 for 21 days. In Fig 2A, ALP staining on days 14 and 21 revealed the absence of aggregates or nodules in the SAMP6 osteoblasts that were obvious in the SAMR1 osteoblasts, indicative of decreased osteoblast maturation. By measuring ALP activity, SAMP6 osteoblasts presented (463±33) or (436±51) nm/min/μg protein at day 14 or 21, which is lower than that of SAMR1 osteoblasts (1805±165) nm/min/μg protein at day 14 or (2125±76) nm/min/μg protein at day 21. The staining was accompanied by a decrease in ALP activity (Fig 2B). After Alizarin red staining, mineralized nodules in SAMR1 cultures were observed, whereas the SAMP6 cultures displayed a smaller number of nodules (Fig 2C and 2D). In addition, Runx2, Colla1 and Oc mRNA expression levels in SAMP6 osteoblasts were significantly downregulated compared with those in SAMR1 osteoblasts (Fig 2E). Taken together, osteoblast differentiation is decelerated and inhibited in response to exogenous BMP-2 in SAMP6 osteoblasts compared with that in SAMR1 osteoblasts.

### Icariin promotes proliferation and migration in SAMP6

**Fig 3:**
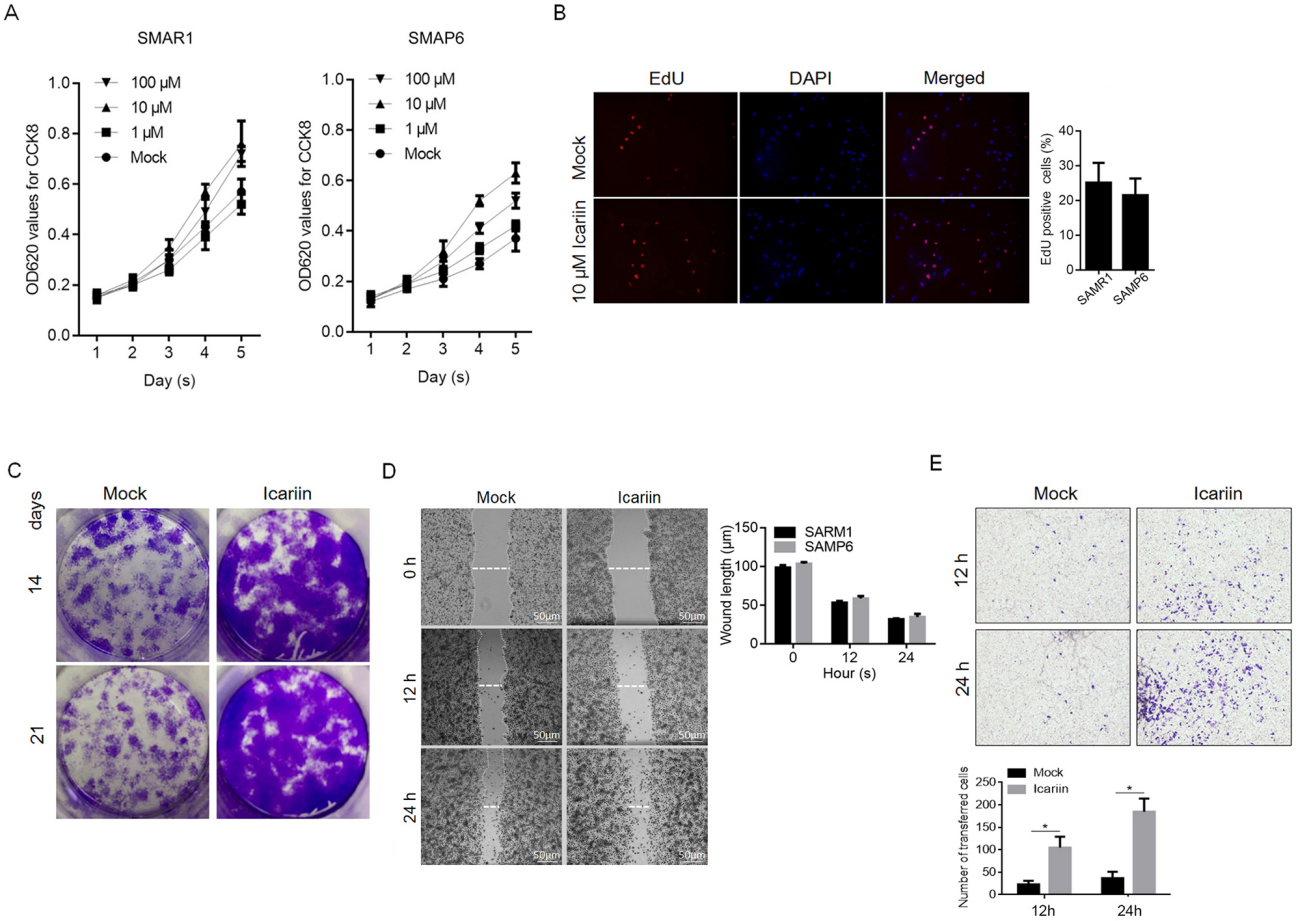
Icariin enhances SAMP6 osteoblast proliferation and migration. After icariin treatment, cell viability (A), cell proliferation (B & C) and migration (D & E) were analyzed in SAMP6 osteoblasts. *P<0.05 vs. Mock group.

Icariin is widely used in traditional Chinese medicine for the treatment of joint and bone disorders, as well as preventing bone loss [9, 28]. It was reported that icariin treatment increases chondrocyte proliferation, which prompted us to assess the effects of icariin on proliferation in SAMP6 osteoblasts. Both SAMR1 and SAMP6 osteoblasts were treated with different concentrations of icariin for 2 days. As shown in Fig 3A, prolonged treatment of icariin at a range of concentration of 1, 10 and 100 μM promoted cell proliferation in both cell types. Icariin treatment achieved the highest viability and was employed for further study. To further confirm the promotion effect of icariin on SAMP6 osteoblast proliferation, we performed the EdU incorporation assay and colony-formation assay. The results showed that 10 μM icariin treatment increased EdU incorporation in SAMP6 osteoblasts, a level that is significantly higher than that in the control group (Fig 3B). The colony formation assay also showed that icariin treatment formed more colonies compared with the control group (Fig 3C).

Osteoblast migration is one of the critical factors for bone maturation and repair [29, 30]. Therefore, we examined the effect of 10 μM icariin treatment on cell migration. SAMP6 osteoblasts treated with 10 μM icariin for 24 hrs. were replated into migration chambers and imaged at 0, 12 and 24 hrs., and the distance traveled was quantified (Fig 3D). Compared with the mock group, treated osteoblasts displayed an increase in the migration distance of approximately 50–70%. For further confirming the effects of icariin on migration, modified transwell assay was performed. Consistent with scratching assay, the number of transferred SAMP6 osteoblasts is significantly larger than that of SAMR1 osteoblasts (Fig 3E).

### Icariin enhances maturation and mineralization in the presence of BMP-2

**Fig 4:**
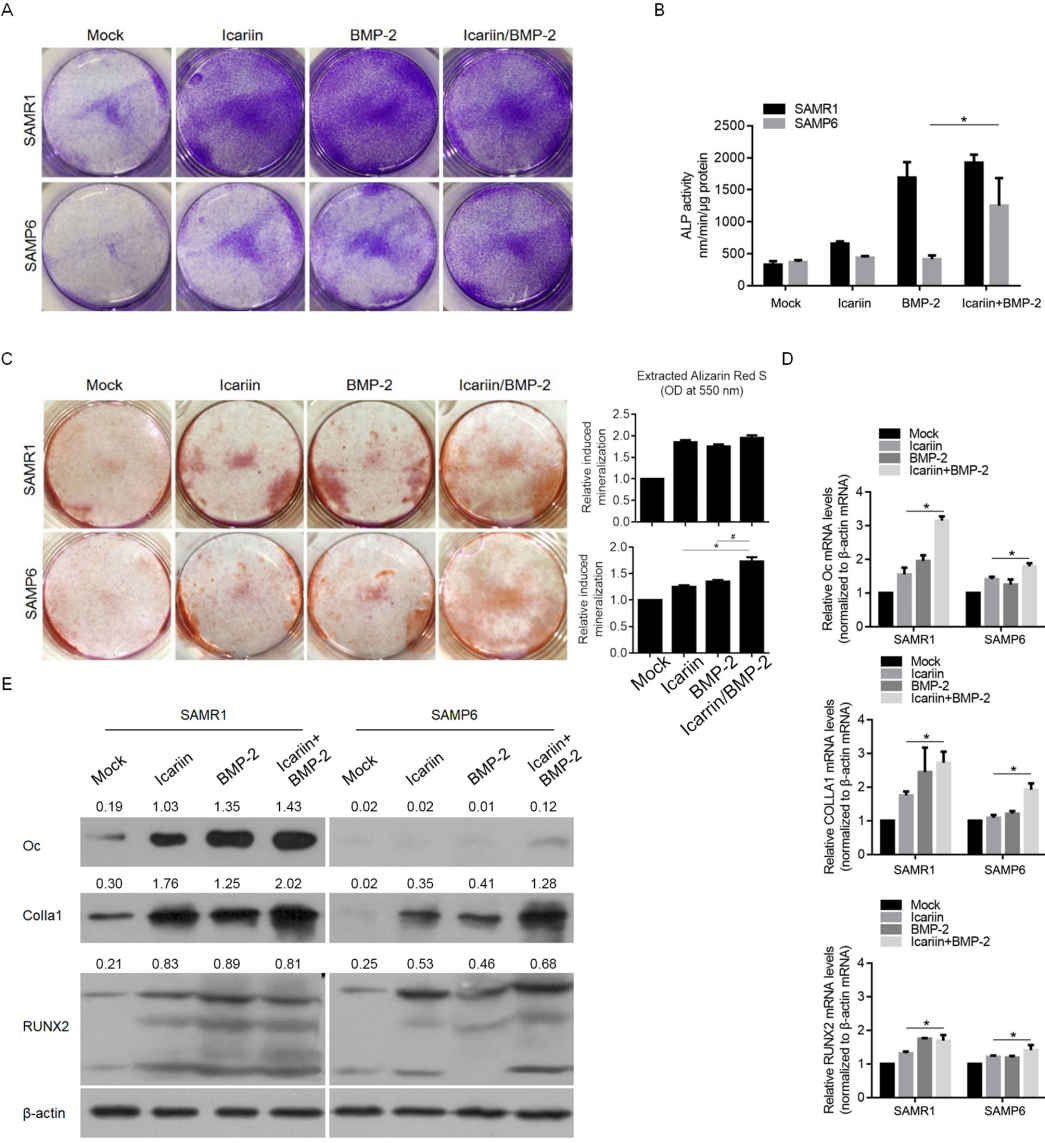
Icariin enhances maturation and mineralization in the presence of BMP-2 compared with SAMR1 osteoblasts. (A) After exposure to 10 μM icariin and BMP-2, ALP staining was performed. (B) ALP activity was measured after 10 μM icariin and BMP-2 treatment. *P<0.05 vs. Icariin group; #P<0.05 vs. BMP-2 group. (C) Alizarin red S staining was performed to detect mineralization. (D) Semiquantitative Western blotting was performed to detect the protein levels of Runx2, collar I and OC after BMP-2 treatment. *P<0.05 vs. Icariin group.

The results that SAMP6 osteoblasts displayed less sensitivity to BMP-2 exposure promoted us to examine the role of icariin in BMP-2-induced SAMP6 osteoblast differentiation. As shown in Fig 4A and 4B, treatment of SAMP6 osteoblasts with 10 μM icariin enhanced BMP-2-induced ALP expression and activity. Interestingly, under normal osteogenic culture conditions without BMP-2, supplemented icariin failed to enhance ALP expression, indicating that its role in promoting differentiation is dependent on the presence of BMP-2 in SAMP6 osteoblasts (Fig 4A and 4B). We also examined the role of icariin in mineralization, which is the final stage of osteoblastic differentiation in vitro. Consistent with its roles in ALP expression and activity, BMP-2 exposure increased the formation of mineralized nodule formation and Icariin treatment enhanced BMP-2-induced nodule formation (Fig 4C). By performing RT-qPCR and semiquantitative Western blot, the results also indicate that icariin improved the upregulation of Oc, Colla1 and Runx2 by BMP-2 induction (Fig 4D and 4E). These results indicate that icariin regulates BMP-2-induced osteoblast differentiation and mature bone nodule formation.

### *CTGF* is potentially a regulating target of icariin in SAMP6 osteoblasts

**Fig 5:**
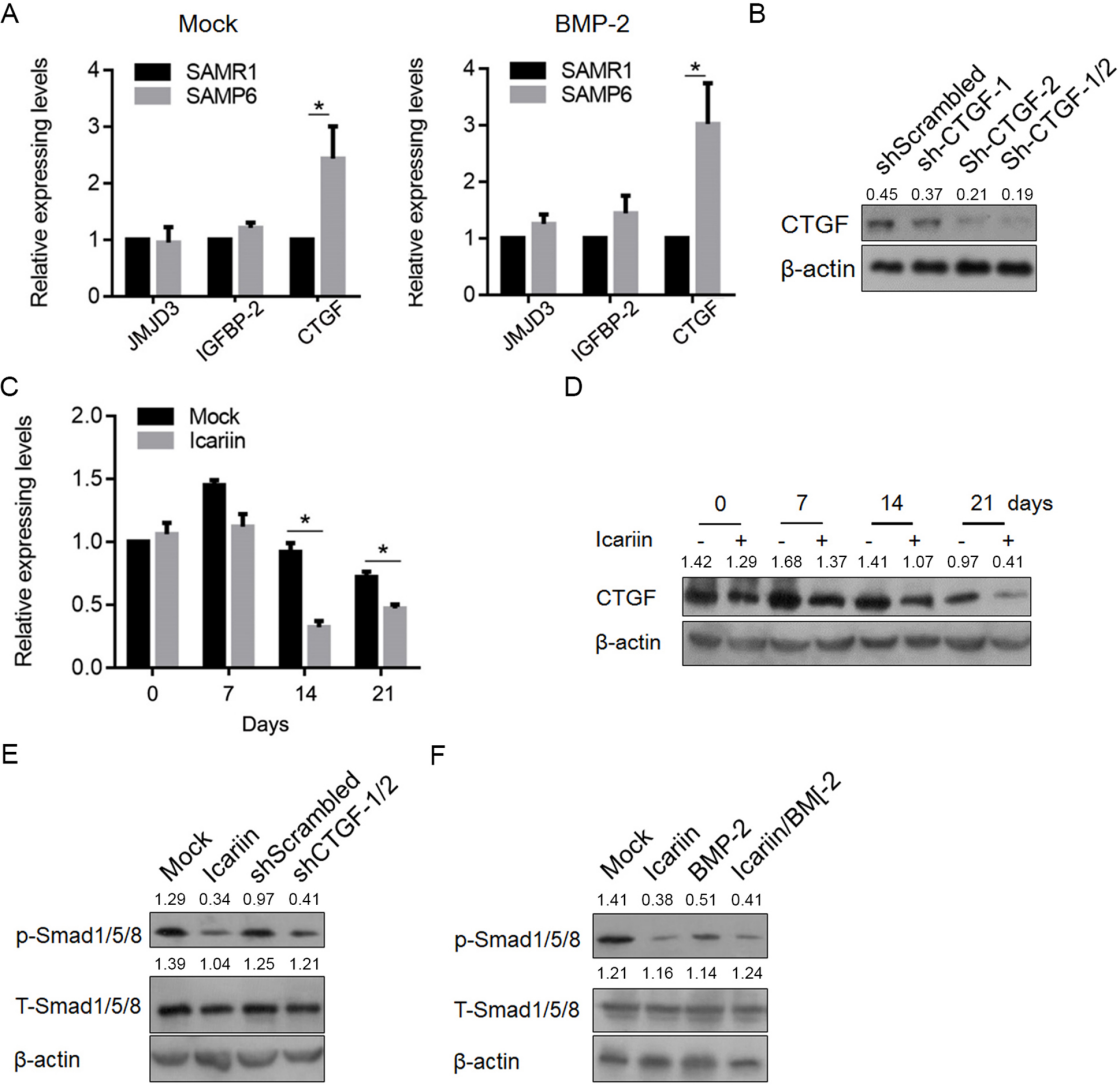
Icariin regulates SAMP6 osteoblasts via transcriptionally regulating *CTGF*. (A) RT-qPCR and semiquantitative Western blotting were performed to measure the JMJD3, IGFBP-2 and *CTGF* expression levels. *P<0.05 vs. SAMR1. (B) Knockdown of *CTGF* by si*CTGF* efficiently decreased the *CTGF* protein levels in SAMP6 osteoblasts. (C) Knockdown of *CTGF* decreased the proliferation of SAMP6 osteoblasts. *P<0.05 vs. mock group. (D) After 7-, 14-, and 21-day exposure to icariin, the expression level of *CTGF* was measured. (E) Icariin exposure activated the phosphorylation of Smad-1/5/8. (F) The effects of icariin exposure and *CTGF* knockdown on SAMP6 osteoblasts were dependent on Smad-1/5/8.

To determine the mechanism by which SAMP6 osteoblasts are insensitive to BMP-2, we assessed the expression levels of JMJD3, IGFBP-2 and *CTGF*, which are reported to be regulated by BMP-2 and critical for osteoblast differentiation [20, 31, 32]. As shown in Fig 5A, compared with the expression in SAMR1 osteoblasts, JMJD3 and IGFBP-2 levels were upregulated but not significantly, and *CTGF* was significantly upregulated in SAMP6 osteoblasts. BMP-2 treatment exerts undetectable effects these three genes (Fig 5A). We transfected siRNA targets to *CTGF* mRNA and confirmed the knockdown efficiency is approximately 70% (Fig 5B) and examined *CTGF*’s effects on proliferation and migration. The results indicated that knockdown of *CTGF* promoted proliferation and migration in SAMP6 osteoblasts (Fig 5C). The effects of icariin on enhancing BMP-2-induced SAMP6 osteoblast differentiation indicate its potential regulation of *CTGF*. By considering this, we measured *CTGF* mRNA levels after icariin treatment for 7, 14 and 21 days. Without disturbing JMJD3 and IGFBP-2 mRNA levels (data not shown), icariin treatment decreased *CTGF* mRNA (Fig 5D). Because Smads 1, 5, and 8 are critical mediators of BMP signaling and are tightly involved in osteoblast differentiation, we investigated whether icariin treatment could be attributed to an increase in the p-Smad 1/5/8 levels. As shown in Fig 5E, both icariin treatment and sh*CTGF* introduction decreased phosphorylated Smad1/5/8. To determine whether *CTGF* expression inhibits BMP-2’s effect on SAMP6 osteoblasts, we treated SAMP6 osteoblasts with icariin/BMP-2, or icariin or BMP-2 alone for 2, 4 and 6 hrs. The p-Smad 1/5/8 protein levels demonstrated an increase in icariin/BMP-2 stimulation in SAMP6 osteoblasts, and BMP-2 alone failed to stimulate an increase in p-Smad 1/5/8 protein levels, indicating the potential mechanism of icariin’s regulatory effect on BMP-2 stimulation via stimulating the phosphorylation of Smad-1/5/8 (Fig 5F).

## Discussion

The senescence-accelerated mouse SAMP6 strain presents several characteristics of senile osteoporosis, such as diminished rates of endosteal bone formation, decreased marrow osteoprogenitors and reduced bone strength compared with age-matched SAMR1 controls [33–37]. In this study, we first isolated SAMP6 and SAMR1 osteoblasts and compared the proliferation, differentiation and mineralization capacities between them. Without displaying detectable morphological difference between SAMP6 and SAMR1 osteoblasts, SAMP6 osteoblasts present decreased cell viability, proliferating capacity and cell cycle arrest at G1/G0 (Fig 1A, 1B and 1C). The increased proportion of G1/G0 and decreased proportion of G2/M potentially resulted the difference of proliferation between SAMR1 and SAMP6. By detecting ALP activity, as well as the levels of osteoblastic marker genes, including RUNX2, Colla1 and Oc, SAMP6 and SAMR1 osteoblasts display normal maturation and mineralization (Fig 1G and 1H), indicating similar osteoblast development and function. Interestingly, in the presence of 100 ng/ml BMP-2, it presents decelerated and inhibited differentiation in response to exogenous BMP-2 in SAMP6 osteoblasts compared with that in SAMR1 osteoblasts. All these data demonstrated that SAMP6 is insensitive to BMP-2 stimulation in an unknown manner.

Icariin is a major active compound isolated from HEF registered in the Chinese pharmacopoeia. By preventing ovariectomy-induced bone loss and decelerated bone formation, icariin is used for the treatment of kidney disease, joint disease, and other disorders. By performing in vitro experiments, icariin could induce osteoblast proliferation, differentiation and mineralization via activating extracellular signal-regulated kinases (ERKs) and the c-Jun N-terminal kinase (JNK) singling pathway [14]. Icariin was reported to inhibit the formation and activation of osteoblasts by suppressing mitogen-activated protein kinases (MAPK)/NF-kB regulated HIF-1α and PGE_2_ synthesis [15, 16]. Icariin also exerts protective effects on vancomycin-induced bone loss and is an effective antibiotic for the treatment of bone infection by exhibiting osteoplastic properties in osteoblasts [17, 38].

JMJD3 is required for osteoblast differentiation and bone formation in mice by transcriptionally regulating RUNX2 in vitro and in vivo [39]. Insulin-like growth factor binding protein two (IGFBP-2) is important for regulating the acquisition of normal bone mass in mice by BMP-2 stimulation [32]. *CTGF* has been shown to regulate osteogenesis after BMP-2 stimulation [20]. For their tight association with BMP-2-induced osteoblast maturation, we evaluated the differences in their factors in SAMP6 and SAMR1 osteoblasts. The results showed that *CTGF* is upregulated in SAMP6 osteoblasts but not JMJD3 or IGFBP-2. *CTGF* is reported to be a negative regulator in BMP-2-induced osteoblast maturation and mineralization [20], indicating its potential role in regulating SAMP6 osteoblast development. By exposure to icariin, SAMP6 osteoblasts, but not SAMR1 osteoblasts, displayed significant downregulation of *CTGF* (Fig 5D), without disturbing the JMJD3 or IGFBP-2 expression levels (data not shown). This demonstrated the specific regulatory role of icariin in *CTGF*-expressing levels. Notably, despite icariin treatment, *CTGF* presented a slow decrease over time (Fig 5F), hinting that, in the process of osteoblast development, reduced *CTGF* levels are necessary and required for osteoblast sensitization to BMP-2. However, the mechanism underlying icariin’s regulatory role regarding *CTGF* remains largely unknown.

SAMP6 is accepted as a model of senile osteoporosis by displaying low bone mass and slow bone loss [40]. At an early age, SAMP6 presents global low bone density [41], marrow osteogenic defects [42], and reduction of the femoral weight and calcium and phosphorus levels [43, 44]. In this study, it was revealed that *CTGF* is highly upregulated in SAMP6 osteoblasts compared with that in SAMR1 osteoblasts, causing the insensitivity of BMP-2. Although the knockdown of *CTGF* by shRNA slightly affected proliferation in SAMP6 osteoblasts, it obviously sensitized SAMP6 osteoblasts to BMP-2 stimulation and promoted osteoblast development and maturation. These results indicate that the abnormality of *CTGF* expression was potentially a key underlying cause of senile osteoporosis by affecting osteoblast development and maturation.

In conclusion, our findings clearly demonstrate the regulatory roles of *CTGF* in BMP-2-induced osteoblast development and maturation. These findings indicate the abnormality of *CTGF* expression as a novel mechanism of senile osteoporosis by reducing osteoblasts. Based on the observations made in the current study, we found that icariin treatment significantly decreased *CTGF* expression and promoted BMP-2-induced osteoblast development and maturation. Under normal conditions, icariin slightly affects osteoblast differentiation. In the BMP-2-inducing condition, icariin treatment downregulated *CTGF* expression and promoted BMP-2-induced osteoblast differentiation, indicating its potential therapeutic effects on *CTGF* abnormality-induced BMP-2 insensitivity.

## Supporting information

S1 File ARRIVE guidelines checklist. (DOCX)

S2 File Blot and images. (RAR)
