# Supplementary material for: Flavonoid compound icariin enhances BMP-2 induced differentiation and signalling by targeting to connective tissue growth factor (CTGF) in SAMP6 osteoblasts
Source: PLoS One. 2018 Jul 10;13(7):e0200367. doi: 10.1371/journal.pone.0200367 (PMC6039035; doi:10.1371/journal.pone.0200367)
Supplement: S1 File — (DOCX) [file pone.0200367.s001.docx]

**Title**:

Flavonoid compound Icariin enhances BMP-2 induced differentiation and signalling by targeting to Connective tissue growth factor (CTGF) in SAMP6 osteoblasts

**Abstract**:

*Background* Icariin, a major active flavonoid glucoside, is widely used for the treatment of bone injury and rebuilding in the clinic because of its roles in suppressing osteoblastogenesis and promoting osteogenesis. The senescence-accelerated mouse SAMP6 was accepted as a useful murine model to reveal the mechanism of senile osteoporosis and the therapeutic mechanism of drug activity. However, little is known about the characteristics of SAMP6 osteoblasts and the associated regulatory roles of icariin.

*Methods* We isolated and cultured osteoblasts from SAMP6 or SAMR1 mice and compared their proliferation, migration, and differentiation by performing the CCK-8 assay, cell counting assay, EdU staining, cell cycle analysis, ALP staining and activity measurement, Alizarin red staining, and RT-qPCR analysis to measure the levels of osteoblast markers, including RUNX2, Colla1 and Oc. To assess the effects of icariin on BMP-2-induced osteoblast differentiation, after BMP-2 treatment, osteoblast markers were analyzed by RT-qPCR and semi-quantitative Western blotting. The effects of icariin on connective tissue growth factor (CTGF) were measured by RT-qPCR. shRNA targeting CTGF mRNA was employed to knockdown its expression level in osteoblasts.

*Results* The SAMP6 osteoblasts presented decreased the development and differentiation activity compared with SAMR1 osteoblasts, indicating that they are the potential mechanisms underlying age-associated disease. Moreover, SAMP6 osteoblasts presented upregulated CTGF compared with SAMR1 osteoblasts. Icariin enhanced BMP-2-induced osteoblast differentiation by downregulating CTGF expression, which tightly regulates osteoblast differentiation. By downregulating CTGF, icariin treatment upregulated phosphate-Smad1/5/8, indicating its activating effects on the BMP signaling pathway.

*Conclusion* These results suggest that decreased osteoblast development and function potentially contributes to age-associated disease. Icariin exerts enhancing effects on BMP-2-mediated osteoblast development via downregulating CTGF.

Background:

Senescence-accelerated mice (SAM) are a series of inbred stains developed from the AKR/J strain consisting of 4 senescence-resistant strains (SAMR) and 9 senescence-prone strains (SAMP) [1, 2]. Compared with the SAMR strains, which present normal senescence, SAM-P6 (SAMP6) strains exhibit accelerated-senescence phenotypes by developing osteoporosis within a few months of birth [3], increased numbers of mature adipocytes (Aps) within the bone marrow [4] or decreased osteoblastogenesis [5], and they have been accepted as an experimental animal model to research age-associated disease [6], including being used as a murine model of senile osteoporosis. By employing the SAMP6 model, O’Sullivan and colleagues found that one of the main factors contributing to the accelerated loss of bone mass in SAMP6 is the presence of impaired osteoblast progenitors affecting proliferation and cell viability in SAMP6 bone marrow, indicating the potential mechanism of senile osteoporosis [7]. However, little is known about the contribution of SAMP6 osteoblasts to the age-associated phenotype.

SAMP6 exhibit accelerated-senescence phenotypes. The difference of physiological processes of osteoblasts potentially results to the different phenotypes compared with osteoblasts obtained from SAMR mice. For this reason, we studied the physiological difference between osteoblasts obtained from SAMP6, compared with that of SMAR1.

**Relative reference:**

[1] Higuchi K. Genetic characterization of senescence-accelerated mouse (SAM). Exp Gerontol. 1997; 32: 129-138. PMID: 9088910.

[2] Takeda T, Hosokawa M, Higuchi K. Senescence-accelerated mouse (SAM): a novel murine model of senescence. Exp Gerontol. 1997; 32: 105-109. PMID: 9088907.

[3] Takahashi K, Tsuboyama T, Matsushita M, Kasai R, Okumura H, Yamamuro T, Okamoto Y, Toriyama K, Kitagawa K, Takeda T. Modification of strain-specific femoral bone density by bone marrow-derived factors administered neonatally: a study on the spontaneously osteoporotic mouse, SAMP6. Bone Miner. 1994; 24: 245-255. PMID: 8019211.

[4] Kajkenova O, Lecka-Czernik B, Gubrij I, Hauser SP, Takahashi K, Parfitt AM, Jilka RL, Manolagas SC, Lipschitz DA. Increased adipogenesis and myelopoiesis in the bone marrow of SAMP6, a murine model of defective osteoblastogenesis and low turnover osteopenia. J Bone Miner Res. 1997; 12: 1772-1779. PMID: 9383681.

[5] Lecka-Czernik B, Gubrij I, Moerman EJ, Kajkenova O, Lipschitz DA, Manolagas SC, Jilka RL. Inhibition of Osf2/Cbfa1 expression and terminal osteoblast differentiation by PPARgamma2. J Cell Biochem. 1999; 74: 357-371. PMID: 10412038.

[6] Takeda T, Matsushita T, Kurozumi M, Takemura K, Higuchi K, Hosokawa M. Pathobiology of the senescence-accelerated mouse (SAM). Exp Gerontol. 1997; 32: 117-127. PMID: 9088909.

[7] O’Sullivan RP, Greenberger JS, Goff J, Cao S, Kingston KA, Zhou S, Dixon T, Houghton FD, Epperly MW, Wang H, Glowacki J. Dysregulated in vitro hematopoiesis, radiasensitivity, proliferation, and osteoblastogenesis with marrow from samp6 mice. Exp Hematol. 2012; 40: 499-509. PMID: 22326715.

Objectives:

The primary objective is to reveal the characteristics of SAMP6 osteoblasts compared with that of SMAR1. Meanwhile, the therapeutic effects of icariin on development of osteoblasts is also be researched.

Ethical statement

All animal experiments were approved by the Wenzhou Traditional Chinese Medicine Hospital Ethics Committee.

Study design

SAMP6 and SMAR1 mice were obtained.

The experimental unit is a single of animal.

| Experimental procedures |
| --- |

New born SAMP6 and SAMR1 mice were bought from Chengdu Dashuo Experimental Animal Research Center (Chengdu, China) and were allowed to be free access to food and water and kept under standardized environmental conditions (12h light/dark cycle, 24℃±1℃ and 55%±1% relativehumidity). Animals were sacrificed to isolate primary osteoblasts from parietal calvaria pieces in accordance with the Guide for the Care and Use of Laboratory Animals. Briefly, after being anaesthetized using combination of 100mg/kg ketamine and 25mg/kg xylazine, and then decapitated, calvaria pieces were placed in digestion media containing 0.1% Collagenase (Sigma–Aldrich, St. Louis, MO, USA)/1% trypsin (Life Technologies, Grand Island, NY, USA) for 30 min at 37℃. Digested supernatant was centrifuged at 400g, 4℃ for 10 min and cells were resuspended with DMEM (Life Technologies) supplemented with 10% fetal bovine serum (FBS; Life Technologies) and plated in 100 mm dishes (Corning Incorporated Life Science, NY, USA). For Icariin (Chinese National Institute for Control of Pharmaceutical and Biological Products, Beijing) treatment, 10^-7^, 10^-6^, or 10^-5^ M of Icariin was supplemented to medium for 7, 14, or 21 days. For BMP-2 stimulation, recombinant BMP-2 (R&D systems, Minnesota, USA) was reconstituted to a concentration of 10μg/ml in PBS containing 0.1% BSA and stored at -20℃. For stimulating differentiation, the final concentration of BMP-2 (100ng/ml) was used every three days. For evaluating levels of p-Smad 1/5/8, cells were pre-cultured in 0.1% FBS for 24 hrs. and then stimulated with BMP-2 for 2, 4, or 6 hrs.

Experimental animals

The animals used are SAMP6 and SAMR1 mice. 4 week-old, female mice were employed.

Housing and husbandry

Animals were allowed to be free access to food and water and kept under standardized environmental conditions (12h light/dark cycle, 24℃±1℃ and 55%±1% relative humidity). Before sacrifice, animals were anaesthetized using combination of 100mg/kg ketamine and 25mg/kg xylazine to minimize hurt.

Sample size

For each strain, 2 animals were used.

| Allocating animals to experimental groups |
| --- |

None

Experimental outcomes

Our results suggest that decreased osteoblast development and function potentially contributes to age-associated disease. Icariin exerts enhancing effects on BMP-2-mediated osteoblast development via downregulating CTGF.

Statistical methods

None

Baseline data

None

| Numbers analysed |
| --- |

None

| Outcomes and estimation |
| --- |

None

| Adverse events  None |
| --- |

Interpretation/scientific implications

None

Generalisability/ translation

None

| Funding |
| --- |

The study was supported by Natural Science Foundation of Zhejiang Province. Award Number: LY16H270015. Recipient: Xiaofeng Wang.
